# Sleep disturbances are associated with irritability in ASD children with sensory sensitivities

**DOI:** 10.1186/s11689-023-09491-z

**Published:** 2023-07-21

**Authors:** Alona Molcho-Haimovich, Liat Tikotzky, Gal Meiri, Michal Ilan, Analya Michaelovski, Hen Schtaierman, Hava M. Golan, Yair Sadaka, Idan Menashe, Ilan Dinstein

**Affiliations:** 1 Department of Psychology, Ben-Gurion University of the Negev, Beer Sheva, Israel; 2 The Azrieli National Centre for Autism and Neurodevelopment Research (ANCAN), Ben Gurion 1, Beer-Sheva, Israel; 3 Pre-School Psychiatry Unit, Soroka University Medical Center, Beer Sheva, Israel; 4 Zusman Child Development Center, Soroka University Medical Center, Beer Sheva, Israel; 5 Child Development Center, Maccabi Health Services, Beer Sheva, Israel; 6 Department of Physiology and Cell Biology, Faculty of Health Sciences, Ben Gurion University of the Negev, Beer Sheva, Israel; 7 Child Development Center, Ministry of Health, Beer Sheva, Israel; 8 Public Health Department, Ben-Gurion University of the Negev, Beer Sheva, Israel; 9 Cognitive & Brain Sciences Department, Ben-Gurion University of the Negev, Beer Sheva, Israel

## Abstract

**Background:**

Parent reports suggest that 44–84% of children with ASD exhibit sleep disturbances that are of clinical concern. Previous studies have reported that, in children with ASD, the severity of sleep disturbances is associated with the severity of either sensory problems or aberrant behaviors, but none have performed combined analyses with measures of both sensory and aberrant behaviors symptom domains from the same children.

**Methods:**

We examined parent reports of 237 children with ASD, 1.4–8.7 years old, using the child sleep habits questionnaire (CSHQ), sensory profile (SP), and aberrant behaviors checklist (ABC).

**Results:**

The analyses revealed that sleep disturbances were most strongly associated with SP sensory sensitivity and ABC irritability scores. Together these scores explained 35% of the variance in total CSHQ scores. Moreover, sensory sensitivity scores moderated the association between irritability and sleep disturbances, indicating that sleep disturbances were significantly associated with irritability only in children with moderate to severe sensory sensitivities.

**Conclusion:**

We suggest that the three symptom domains may interact and exacerbate each other such that successful intervention in one symptom domain may have positive impact on the others. Further intervention studies testing this hypothesis are highly warranted.

## Introduction

Sleep disturbances, sensory problems, and aberrant behaviors are common in many children with Autism Spectrum Disorder (ASD) and cause considerable distress for both children and their parents [22, 24, 30]. To date, studies have examined the relationship between sleep disturbances and sensory problems or the relationship between sleep disturbances and aberrant behaviors, independently of each other in different cohorts. The goal of this study was to determine whether sleep disturbances are associated with specific sensory problems and specific aberrant behaviors within a single cohort and identify potential interactions.

According to parent reports, sleep disturbances are evident in 44–84% of ASD children in contrast to 10–30% of typically developing (TD) children [20, 31, 46, 48], and 30–77% of children with other neurodevelopmental disorders [11]. Studies based on parental reports demonstrate that the most common sleep disturbances in children with ASD include long sleep onset latencies (SOL), frequent night awakenings, short sleep duration, and early morning awakening [27, 48]. Actigraphy studies have reported that children with ASD exhibit prolonged SOL [2, 41], longer nocturnal wake episodes, and shorter sleep duration [27, 54] than matched TD participants. Similarly, polysomnography studies have reported prolonged SOL, shorter sleep duration by 30–40 min [16, 41], reduced amounts of Rapid Eye Movement (REM) sleep [8], and weaker slow wave activity in children with ASD compared to matched TD participants [5].

According to parent reports, sensory problems are also more common in children with ASD (42–95%) and in children with other neurodevelopmental disorders, such as ADHD (40–60%) [42] than in TD children (3–13%) [1, 6, 55] and are highly heterogeneous. Problems may include hyper- and/or hypo-reactivity to different sensory stimuli including unusual interests in some sensory stimuli (e.g., smelling or tasting items) and/or adverse responses to others. These sensory problems are now defined as one of the diagnostic criteria for ASD within the restricted and repetitive behaviors (RRB) domain of the DSM-5 [4]. Sensory problems are associated with sleep disturbances in children with ASD [32, 35, 38]. In particular, hypersensitivity to tactile and auditory stimuli are associated with sleep problems [47, 56, 57]. Moreover, longitudinal changes in sleep disturbances over 1–2 years are correlated with changes in sensory sensitivities such that children with ASD who improve in one symptom domain are likely to also improve in the other [35].

Aberrant behaviors including irritability, aggression, hyperactivity, impulsivity, non-compliance, and self-injury are also common in children with ASD, as reported by their parents [14, 28]. These behaviors generate considerable distress for both the children and their families [13, 28]. Several studies have reported that children with ASD who have sleep disturbances are more likely to exhibit aberrant behaviors than children with ASD who do not have sleep disturbances [15, 22, 34, 36, 46, 52].

To date, studies have examined the relationship between sleep problems and sensory profile or behavioral problems separately. The uniqueness of this study is the examination of the triadic links between these three domains. Moreover, the goal of this study is to examine whether sleep disturbances are associated with specific sensory problems and specific aberrant behaviors within a single cohort and identify potential interactions.

In the present study, we utilized data from an ASD cohort at the Azrieli National Centre for Autism and Neurodevelopment Research (ANCAN) in Israel where parents of ASD children completed sleep disturbances, sensory problems, and aberrant behaviors questionnaires. In line with previous studies described above, we hypothesized that we would find significant correlations between sleep disturbance scores and sensory problem or aberrant behavior scores. Since sensory hypersensitivities are particularly associated with sleep disturbances [47, 56, 57] and are likely to make individuals more irritable, we also hypothesized that we may find an interaction between these symptom domains in the prediction of sleep disturbances.

## Methods

### Participants and design

We analyzed data from 237 Jewish children with ASD that were extracted from the ANCAN database [9, 40]. Children were 1.4–8.7 years old (Mean = 4.4, SD = 1.49) at the time of data collection and 183 (77%) were male (see Table 1). This sample included all children who were diagnosed with ASD between 2019 and 2021 at Soroka University Medical Center and whose parents completed the Child Sleep Habit Questionnaire (CSHQ, [44, 45]), Sensory Profile (Dunn 2014) [10], and Aberrant Behavior Checklist (ABC, [3]). Parents completed all three questionnaires within 6 months of each other. All children fulfilled DSM-5 criteria for ASD as determined by a developmental psychologist and a child psychiatrist or pediatric neurologist. All children completed an ADOS-2 assessment [33] and 83% (197 of the 237 children) also completed a cognitive assessment (see below). None of the children in the current study had a clinical diagnosis of epilepsy or known genetic syndromes. This study was approved by the Soroka University Medical Center Helsinki committee.

**Table 1:** Sample characteristics

	Mean	SD	Min	Max
**Age**	4.41	1.49	1.38	8.71
**CSHQ**				
Total sleep disturbance score	47.1	9.33	33	81
**ABC sub-scales**				
Irritability	11.61	10.27	0	44
Social withdrawal	9.18	8.81	0	43
Stereotype behavior	4.75	5.22	0	20
Hyperactivity	14.92	11.92	0	47
Inappropriate speech	3.27	3.12	0	12
**Sensory Profile sub-scales**				
Sensory seeking	0.27	0.92	-2	2
Sensory avoiding	0.59	0.96	-2	2
Sensory sensitivity	0.64	0.97	-2	2
Registration	0.48	1.02	-2	2
**ADOS scores**				
Total Calibrated Severity Score (CSS)	5.68	2.80	1	10
Social Affect (SA) CSS	5.70	2.84	1	10
Restricted Repetitive Behaviors (RRB’s) CSS	6.29	2.56	1	10
**Cognitive scores** (*n* = 197)	82.71	21.32	47	140

*CSHQ* Children’s Sleep Habit Questionnaire, *ABC* Aberrant Behavior Checklist questionnaire

### Measures

#### Children’s Sleep Habits Questionnaire (CSHQ) [44, 45]

The CSHQ is a parent-report sleep screening questionnaire containing 33 questions that assess sleep disturbances in children. In addition to a total sleep disturbances score, this questionnaire yields scores in eight subscales: (1) bedtime resistance; (2) sleep-onset delay; (3) sleep duration; (4) sleep anxiety; (5) night-waking; (6) parasomnias; (7) sleep-disordered breathing; and (8) daytime sleepiness [44, 45]. The CSHQ has been used with toddlers and preschool aged children [17]. A total sleep disturbance score of 41 is often used as a cutoff for clinical concern [26].

#### Aberrant Behavior Checklist (ABC, [3])

The ABC is a 58-item parent questionnaire that estimates the presence of aberrant behaviors in five subscales: (1) irritability; (2) social withdrawal; (3) stereotypical behavior; (4) hyperactivity; and (5) inappropriate speech. Although the ABC was designed for use in the intellectual and developmental disabilities adult population, it has also been validated for use with children with ASD [25]. Population norms have not been published for this tool.

#### Infant/Child Sensory Profile (SP) (Dunn 2014 [10])

This parent-report questionnaire estimates sensory processing difficulties in four domains: (1) sensation seeking; (2) sensation avoiding; (3) sensory sensitivity; and (4) low sensory registration. The Infant SP questionnaire was used with children ≤ 35 months old (*n* = 48), and the Child SP questionnaire (*n* = 189) was used with children ≥ 36 months old. The SP raw scores are transformed into standardized scores according to population norms [10], which allows individual quantification of relative sensory problems while accounting for the child’s age.

#### ADOS-2

The ADOS-2 is a semi-structured, standardized assessment for measuring ASD symptom severity [33]. Participants in this study completed either the toddler module (*n* = 3), module 1 (*n* = 95), module 2 (*n* = 84) or module 3 (*n* = 60) of the ADOS-2, according to their age and language abilities. ADOS-2 scores of different modules can be compared by transforming raw scores into calibrated severity scores (CSS), which quantify the severity of core ASD symptoms regardless of age and language abilities [12, 18, 21].

#### Cognitive assessments

We administered the Bayley scales of infant and toddler development, 3^rd^ edition [7] with children 1–4.5 years old (*n* = 52; [7]), Wechsler preschool and primary scale of intelligence (*WPPSI-III*) with children 2.6–7.1 years old (*n* = 79; [59]), and the Mullen scales of early learning with children 1.2–5.9 years old (*n* = 66; MSEL:AGS; [43]). The cognitive test was selected by certified developmental psychologists according to the age of the child and the psychologist’s preference. All three tests yield equivalent standardized scores with a mean of 100 and a standard deviation of 15. We combined scores from the three testes given that there are strong correlations across them [7, 29]. Cognitive assessments were successfully completed with 197 of the 237 children. In the remaining children cognitive assessment were unsuccessful due to challenging behaviors that interfered with completing a valid cognitive assessment. Note that such challenges are not exclusive to ASD children.

### Statistical analyses

All statistical analyses were conducted in R-Studio (version 1.1.463). Associations between pairs of measures were tested by computing Pearson correlation coefficients. We also performed a multiple regression analysis with the sleep disturbance scores as the dependent variable and the ABC and SP subscale scores as independent variables (i.e., predictors). Age was also included in the regression model as a control variable. A simple slope analysis was conducted to test whether sensory sensitivity scores moderated the relationship between sleep disturbances and irritability.

## Results

**Fig. 1:**
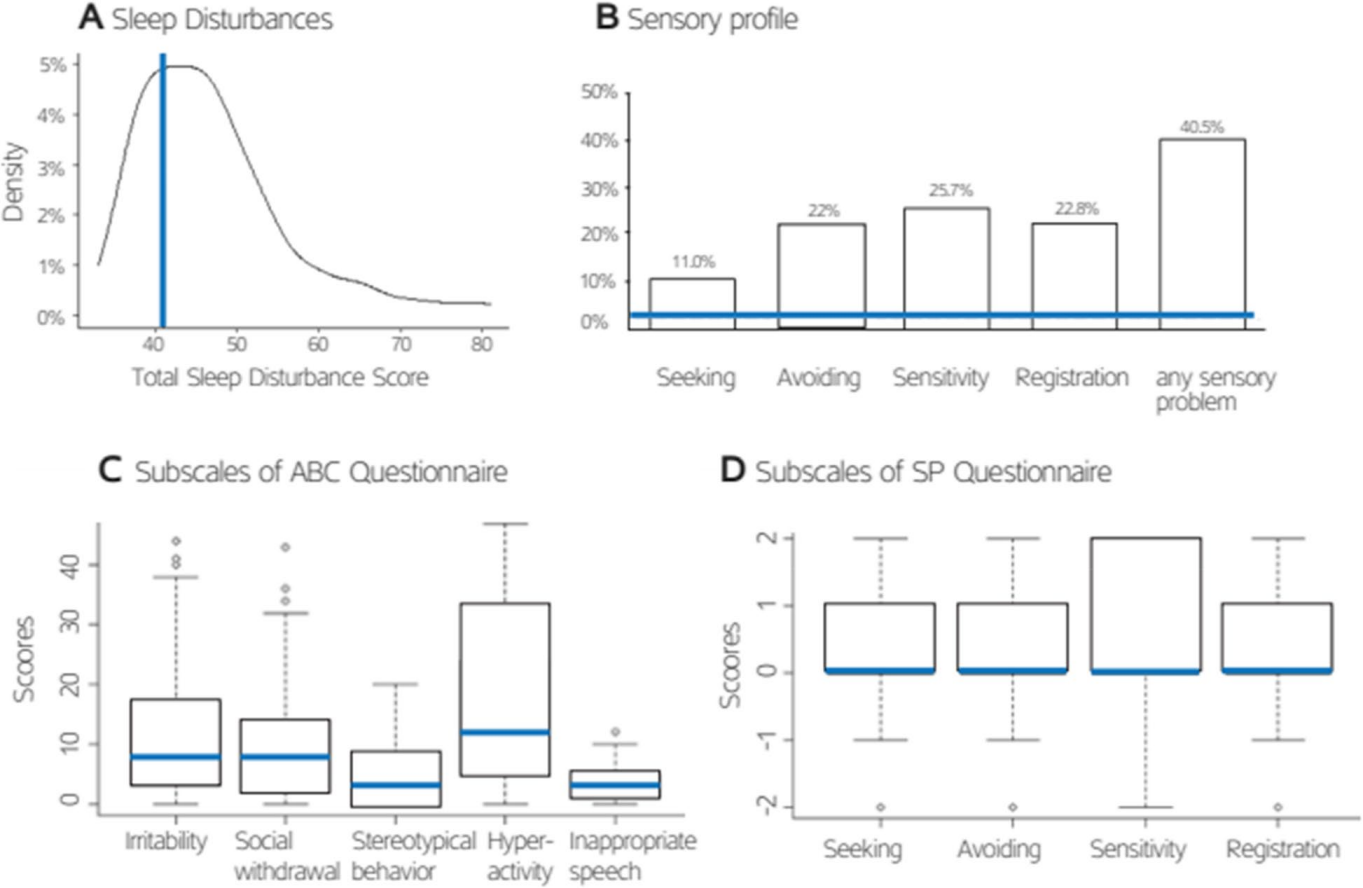
Overview of the sleep disturbances, aberrant behaviors and sensory problems in the examined cohort. **A** Probability density function of CSHQ total sleep disturbance scores. Vertical dashed line: cutoff of 41 indicative of clinically significant sleep disturbances. **B** Percentage of children with Sensory Profile scores that were ≥ 2 standard deviations above the general population mean. Horizontal line: expected percentage of individuals in the general population with scores of 2 standard deviations or above assuming a normal distribution. **C** Box plot figures of the five ABC subscale scores: irritability, social withdrawal, stereotypical behavior, hyperactivity, and inappropriate speech. **D** Box plot figures of the four SP subscale scores: sensation seeking, sensation avoiding, sensory sensitivity, and low registration. Bold line: median of each distribution

Children with ASD exhibited heterogeneous CSHQ total sleep disturbances scores (Fig. 1A) with 70% (166 of 237) exceeding a clinical cutoff of 41 as originally proposed for 4–10 year old TD [44, 45] and ASD [26] children. The children also exhibited heterogeneous SP scores (Fig. 1B) with > 40% of the children exhibiting scores that were ≥ 2 standard deviations above the population norm in at least one SP subscale (Fig. 1B). Note that the most prevalent sensory problem was in the sensory sensitivity domain, indicating hypersensitivity to stimuli, reported for > 25% of the children. Heterogeneous scores were also apparent across children in all 5 ABC subscales (Fig. 1C). Since population norms and clinical cutoffs are not available for the subscales of this questionnaire, it is not possible to quantify the percent of ASD children with abnormal aberrant behaviors.

### Correlations across different symptom domains

CSHQ scores were not significantly correlated with either ADOS total CSS (*r(239)* = 0.04, *p* = 0.52), ADOS social affect (SA) CSS (*r(235)* = 0.050, *p* = 0.44), or ADOS restricted and repetitive behaviors (RRB) CSS (*r(239)* = 0.004, *p* = 0.95). However, there was a marginally significant negative correlation between the CSHQ score and the cognitive scores of the children (*r(195)* = -0.14, *p* = 0.049), indicating that parent reported sleep disturbances explained almost 2% of the variance in cognitive scores across children. Note that we present results without correction for multiple comparisons to increase sensitivity. The correlation between CSHQ and cognitive scores is not significant when applying Bonferroni correction for these 4 comparisons.

**Fig. 2:**
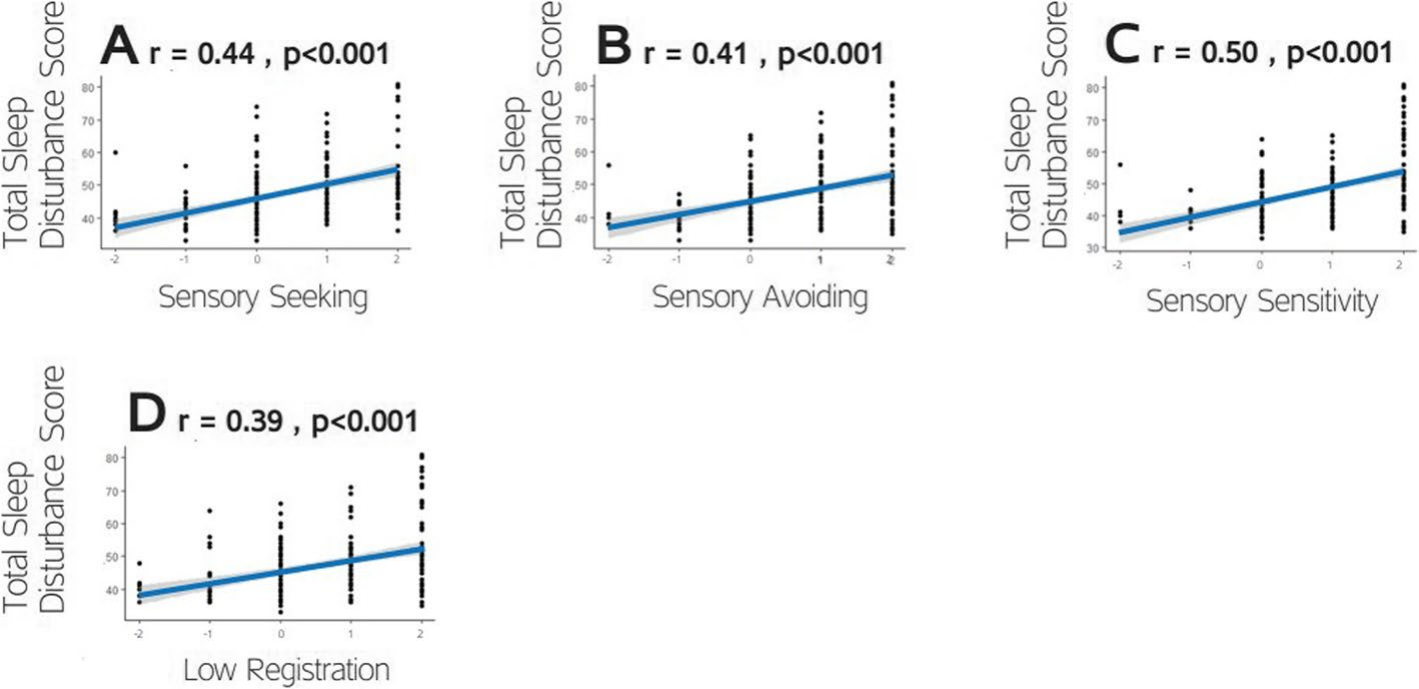
Scatter plots demonstrating the correlations between CSHQ and SP scores for each of the four domains: Sensory Seeking. Sensory Avoiding, Sensory Sensitivity, and Low Registration. Each point represents a single child. Line: linear squares fit. Pearson’s correlation coefficients and corresponding *p* values are noted on each panel

**Fig. 3:**
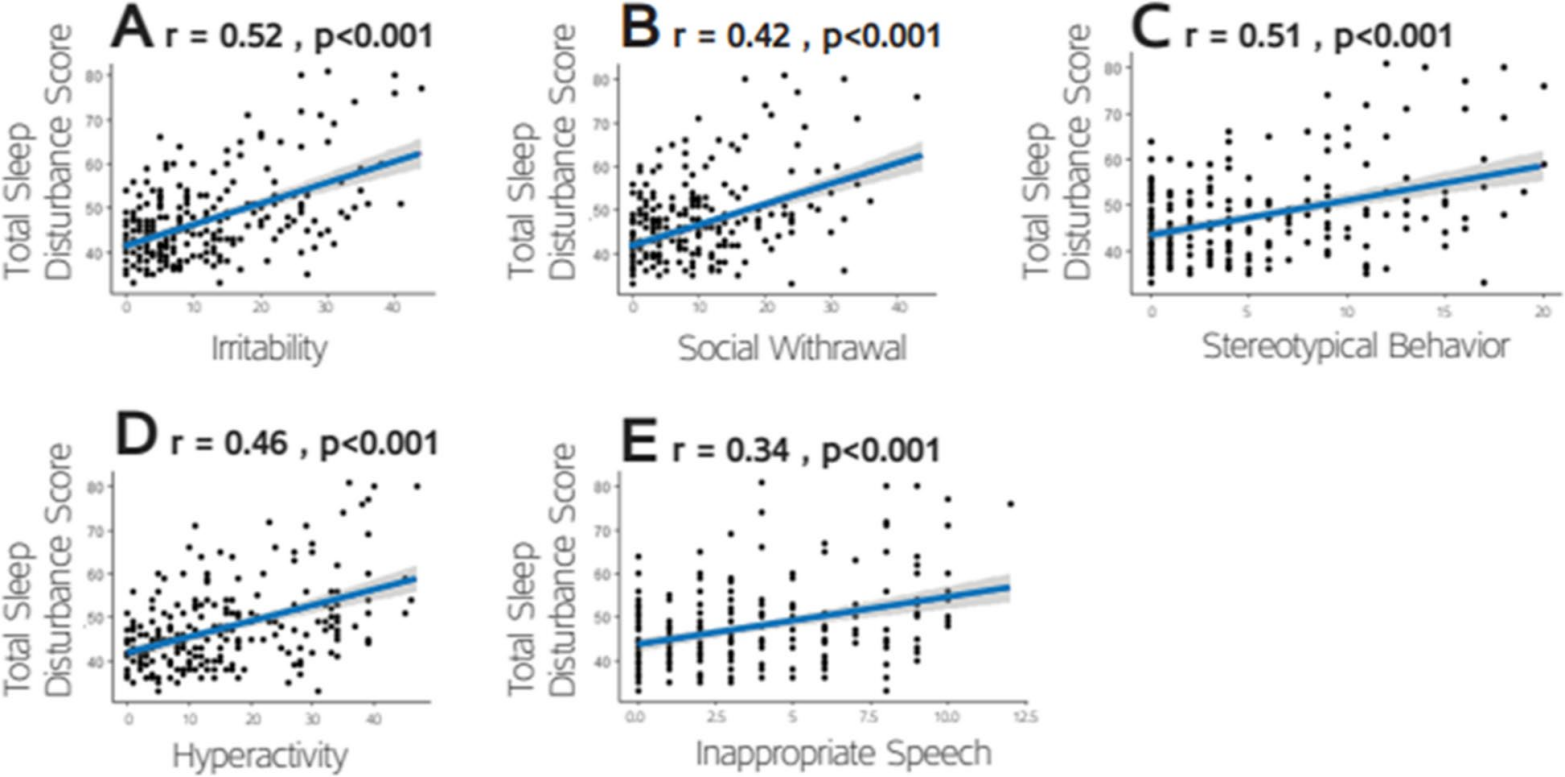
Scatter plots demonstrating the correlations between CSHQ and ABC scores for each of the five subscales: **A** Irritability. **B** Social Withdrawal. **C** Stereotypical Behavior. **D** Hyperactivity. **E** Inappropriate Speech. Each point represents a single child. Line: linear squares fit. Pearson’s correlation coefficients and corresponding *p* values are noted on each panel

In contrast, CSHQ scores were significantly positively correlated with all four SP subscale scores (Fig. 2): Sensation Seeking (*r(235)* = 0.43, *p* < 0.001), Sensation Avoiding (*r(235)* = 0.4, *p* < 0.001), Sensory Sensitivity (*r(235)* = 0.5, *p* < 0.001) and Low Registration (*r(235)* = 0.37, *p* < 0.001). Similarly, CSHQ scores were significantly positively correlated with all five ABC subscales scores (Fig. 3): Irritability (*r(235)* = 0.52, *p* < 0.001), Social Withdrawal (*r(235)* = 0.42, *p* < 0.001), Stereotype Behavior (*r(235)* = 0.41, *p* < 0.001), Hyperactivity (*r(235)* = 0.46, *p* < 0.001), and Inappropriate Speech (*r(235)* = 0.34, *p* < 0.001). All correlations remain significant after Bonferroni correction for these 9 comparisons.

There were moderate to strong positive correlations between ABC and SP subscale scores (Table 2). Moreover, strong correlations were apparent across sub-scale scores of each questionnaire separately (Table 2). Note that all correlations were significant also after Bonferroni correction for the 36 presented correlations.

**Table 2:** Correlations across ABC and SP subscales

	Irritability	Social Withdrawal	Stereotypical Behavior	Hyperactivity	Inappropriate Speech	Sensation Seeking	Sensation Avoiding	Sensory sensitivity
**ABC subscales**								
Social Withdrawal	0.67***							
Stereotype Behavior	0.67***	0.68***						
Hyperactivity	0.82***	0.64***	0.69***					
Inappropriate Speech	0.58***	0.50***	0.54***	0.59***				
**SP domains**								
Sensation seeking	0.51***	0.36***	0.43***	0.60***	0.45***			
Sensation avoiding	0.60***	0.46***	0.43***	0.52***	0.40***	0.53***		
Sensory sensitivity	0.60***	0.50***	0.53***	0.55***	0.38***	0.62***	0.70***	
Low registration	0.49***	0.50***	0.47***	0.47***	0.40***	0.56***	0.68***	0.67***

*ABC* Aberrant Behavior Checklist, *SP* Sensory Profile

^***^p < .0001

### No significant differences in symptom severity across boys and girls

No differences were found between boys and girls in any of the symptom domains described above, including sleep disturbance scores, all five ABC subscales, and all SP subscales except for the sensory avoiding domain (t(242) = 3.06, *p* = 0.02), where boys exhibited higher scores. This difference, however, did not survive Bonferroni correction for these 10 comparisons.

### Predicting the severity of sleep disturbances from ABC and SP scores

Regression analyses were used to examine the ability of individual ABC and SP subscale scores to explain the variance in CSHQ scores. We first included all ABC and SP subscale scores and control variables (age, ADOS score and cognitive level) as predictors in a single multiple regression model. This model explained 31% of the variance in CSHQ scores (Table 3).

**Table 3:** Multiple regression analyses predicting the CSHQ total score from the ABC and SP sub-scales. Model 1: When using all ABC and SP sub-scales as well as age, ADOS, and cognitive scores. Model 2: When using only the ABC irritability sub-scale and the SP sensitivity sub-scale as well as their interaction term

	β	*P*
**Model 1:** adjusted *R*^2^ = 0.31		
***ABC***		
Irritability	**2.30**	**< 0.05**
Social withdrawal	1.42	0.16
Stereotypical behavior	0.28	0.78
Hyperactivity	-0.56	0.57
Inappropriate speech	0.66	0.51
**SP**		
Seeking	1.45	0.15
Avoiding	-0.37	0.70
Sensitivity	**2.62**	**< 0.01**
Low registration	-0.77	0.43
Age	-0.56	0.57
ADOS Score	0.98	0.32
Cognitive Score	1.06	0.29
**Model 2:** adjusted *R*^2^ = 0.35		
***ABC***		
Irritability	**3.60**	**< 0.001**
**SP**		
Sensitivity	**5.11**	**< 0.001**
**Interaction**		
Irritability*Sensitivity	**3.53**	**< 0.001**

*ABC* Aberrant Behavior Checklist, *SP* Sensory Profile

**Fig. 4:**
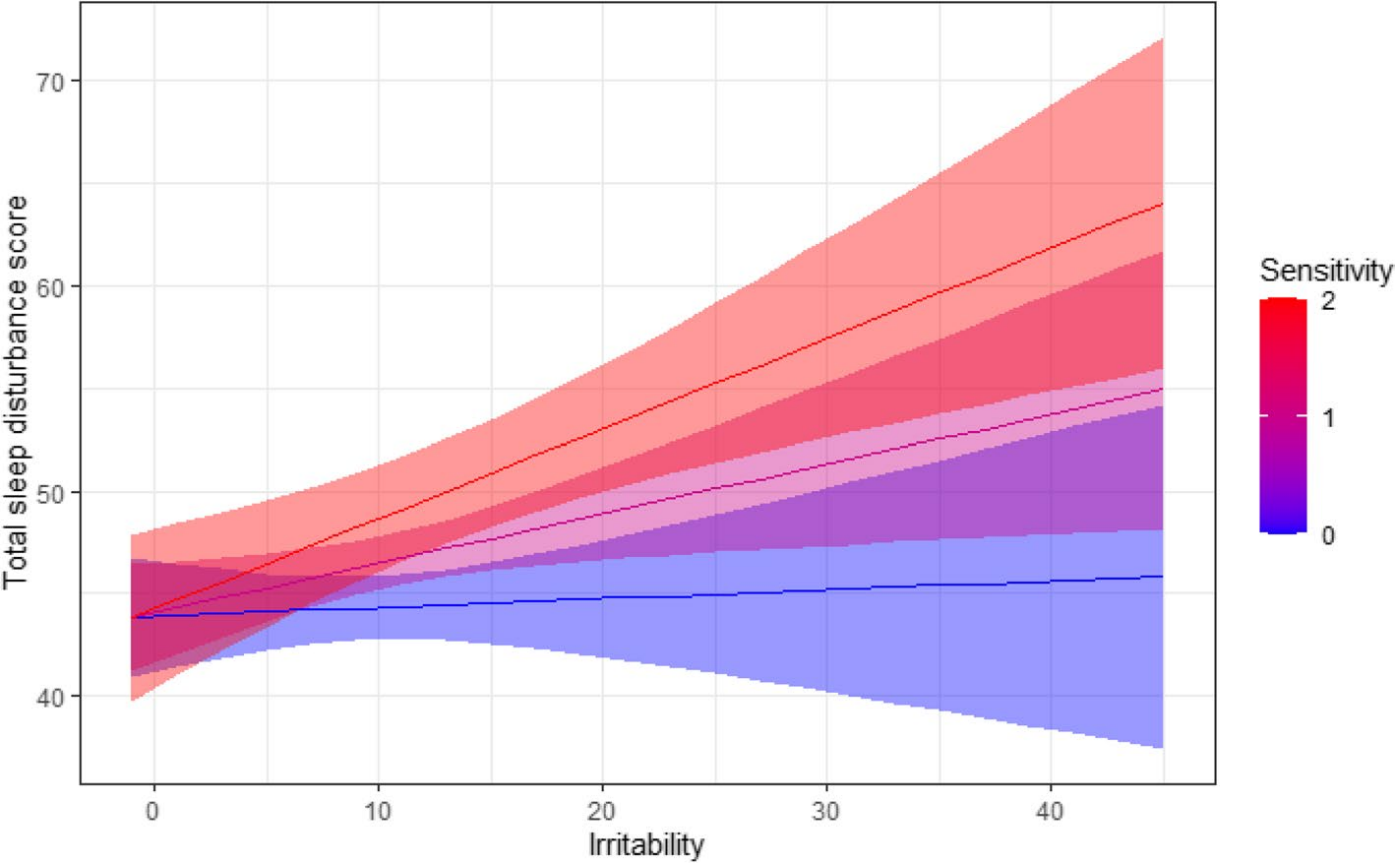
Simple slope analysis demonstrates the moderating effect of sensory sensitivity on the relationship between irritability and CSHQ total scores. Blue line: children with sensory sensitivity scores of 0, -1, or -2. Purple line: children with sensory scores of 1. Red line: children with sensory scores of 2

Of the predictors included in the initial regression model only the sensory sensitivity score from the SP and the irritability score from the ABC reached statistical significance (Table 3). We, therefore, examined another regression model using only irritability and sensitivity as predictors of the CSHQ score while also examining their interaction (Table 3 model 2). The new regression analysis explained 35% of the variance in CSHQ scores. Note that each of these two variables (irritability and sensory sensitivity) single handedly explained 25% of the variance in CSHQ scores in the correlation analyses above (Figs. 3 and 4).

Given the significant interaction between irritability and sensitivity scores in the regression model above, we performed a simple slope analysis. We found that sensory sensitivity moderated the association between irritability and CSHQ scores such that the strength of this association increased with the severity of sensory sensitivities (Fig. 4). The association between irritability and CSHQ scores was strongest for children with sensory sensitivity scores of 2 (B = 0.48, t_237_ = 6.50, p < 0.0001) weaker for children with sensory sensitivity scores of 1 (B = 0.29, t_237_ = 2.18, *p* = *p* < 0.0001), and not significant for children with sensory sensitivity scores of 0 or less (B = 0.11, t_237_ = 1.29, *p* = 0.19).

## Discussion

Our results demonstrate that sleep disturbances in young children with ASD are primarily associated with irritability and sensory sensitivity. Indeed, these two symptoms from the ABC and the SP, respectively, explained 35% of the variance in CSHQ total sleep-disturbance scores when utilized in a regression model. The inclusion of additional scores from other ABC or SP subscales as predictors in the regression model did not increase the variance explained (Table 3). Moreover, sensory sensitivity acted as a moderator for the association between irritability and sleep disturbances, such that irritability was significantly correlated with CSHQ scores only in children with moderate or severe sensory sensitivities (Fig. 4). Taken together, these findings demonstrate that the severity of sleep disturbances in children with ASD is highest in those with severe sensory sensitivities and irritability. We speculate that this relationship is bi-directional, such that the three symptom domains exacerbate each other (e.g., irritability may exacerbate sleep disturbances and vice versa). If this hypothesis is correct, successfully intervening in one symptom domain would lead to improvements in the other domains, as well.

### Prevalence of sleep disturbances, sensory problems and aberrant behaviors

Consistent with previous studies reporting that 44–84% of children with ASD exhibit sleep disturbances [27, 34, 46, 58], 70% of parents in our sample reported sleep disturbance scores that that were higher than proposed clinical cutoffs [44, 45]. Approximately 40% of parents in our sample also reported that their ASD children exhibited sensory problems, with SP scores that were ≥ 2 standard deviations higher than those of the general population. This prevalence is on the lower end of previous reports that have estimated this prevalence at 42–95% of children with ASD [1, 6, 55]. Finally, parents in the current study reported that their ASD children exhibited a variety of aberrant behaviors. However, since there are no population norms for the ABC subscales and no clinical cutoffs, it is not possible to determine the prevalence of aberrant behaviors in our sample.

### Relationship between sleep disturbances and sensory problems

As was reported in previous studies [32, 47, 56, 57], total CSHQ scores were more strongly correlated with sensory sensitivity scores than with scores of other sensory domains on the SP. Moreover, the strength of this specific correlation when using the 2^nd^ edition of the SP (*r* = 0.5, *p* < 0.001) was similar to that reported in a previous study using the 1^st^ edition of the SP (*r* = -0.5, *p* < 0.01, [56, 57]). Note that the 1^st^ edition has a reversed scale where lower scores indicated more severe sensory symptoms, hence the negative correlation.

Some studies have suggested that total CSHQ scores are specifically correlated with hyper-sensitivity in the tactile [56, 57] or auditory [47] domains. Notably, one study demonstrated that correlations between CSHQ and SP sensory sensitivity scores were also apparent in longitudinal changes over a 1–2 year period, indicating that changes in one symptom domain are associated with changes in the other [35]. Although causality cannot be inferred from correlational findings, one possibility is that sensory sensitivity is expressed as increased arousal, which in turn impedes the ability to initiate and maintain sleep [23]. Surprisingly, we have found that girls are more likely to exhibit avoiding behavior, although the effect size was small. Previous studies were limited in their ability to conclude on gender differences in sensory profile and the direction of these differences. Taken together, this finding should be considered with caution.

### Relationship between sleep disturbances and aberrant behaviors

Our findings revealed significant correlations between the total CSHQ scores and all ABC subscales (0.34 < *r* < 0.52, *p* < 0.01), in line with a previous study that reported a significant correlation (*r* = 0.61, *p* < 0.001) between total sleep disturbances on the CSHQ and the sum of aberrant behavior subscales on the ABC [50]. However, other studies have not found significant correlations between CSHQ and ABC subscale scores [19, 58], potentially due to the large heterogeneity of symptoms across different samples. Nevertheless, studies using the CSHQ and alternative parent questionnaires of aberrant behaviors such as the Parental Concerns Questionnaire have also reported significant relationships between sleep disturbances and aberrant behaviors [16, 37], thereby providing additional evidence regarding this relationship.

Among the ABC subscales, irritability was the strongest predictor of total sleep disturbance scores in our results, as also reported in prior studies [39, 50, 51].

### Limitations

An important limitation of the current study is the reliance on parental reports, which are inherently subjective and potentially biased and inaccurate [49]. For example, parents who are generally positively or negatively biased regarding their child’s condition may report higher or lower scores, respectively, on all questionnaire regardless of their specific content. Future studies could, therefore, benefit from the addition of objective sleep measures (e.g., actigraphy) and clinical assessments of aberrant behaviors and sensory problems using, for example, the Sensory Assessment for Neurodevelopmental Disorders [53]. Second, the correlative nature of the study does not allow for the inference of causality, which would require intervening in one of the symptom domains and assessing impact on the other symptom domains. Third, all participants in this study were fluent in Hebrew, which may have limited the participation of minority groups such as Bedouin Arabs and/or Jewish immigrants from Ethiopia or Russia whose primary language is not Hebrew. Fourth, we did not track the identity of the parent who completed the questionnaires, which may have potentially biased the reported scores. Finally, we did not assess the children’s language abilities and, therefore, could not test potential relationships between language delays and the severity of symptoms reported in this study.

## Conclusions

Our results demonstrate particularly strong associations between the severity of sleep disturbances and the severity of irritability and sensory sensitivities as reported by the parents. The structure of these associations is such that irritability was significantly associated with sleep disturbances specifically in ASD children with moderate to high sensory sensitivities. Further studies are warranted for determining whether successful treatment of one symptom domain may impact positively on other symptom domains.

## Data Availability

Data are available through the Azrieli National Centre for Autism and Neurodevelopment Research (ANCAN) and will be shared by request.
